# Malaria prevalence pattern observed in the highland fringe of Butajira, Southern Ethiopia: A longitudinal study from parasitological and entomological survey

**DOI:** 10.1186/1475-2875-10-153

**Published:** 2011-06-07

**Authors:** Solomon Tesfaye, Yeshambel Belyhun, Takele Teklu, Tesfaye Mengesha, Beyene Petros

**Affiliations:** 1 Department of Biology, Faculty of Applied Sciences, University of Gondar, P.O. Box 196, Gondar, Ethiopia; 2 Department of Microbiology, Immunology, and Parasitology, College of Medicine and Health Sciences, University of Gondar, P.O. Box 196, Gondar, Ethiopia; 3 Department of Medical Laboratory Sciences, College of Medicine and Health Sciences, University of Gondar, P.O. Box 196, Gondar, Ethiopia; 4 Ethiopian Health and Nutrition Research Institution, P.O. Box 1242. Addis Ababa, Ethiopia; 5 Department of Biology, School of Graduate Studies, Addis Ababa University, P.O. Box 1176, Addis Ababa, Ethiopia

## Abstract

**Background:**

In Ethiopia, information regarding highland malaria transmission is scarce, and no report has been presented from Butajira highland so far whether the appearance of malaria in the area was due to endemicity or due to highland malaria transmission. Thus this study aimed to determine the presence and magnitude of malaria transmission in Butajira.

**Methods:**

For parasitological survey, longitudinal study was conducted from October to December 2006. The entomological surveys were done from October to December 2006 and continued from April to May 2007. Both parasitological and entomological surveys were done using standard procedures.

**Results:**

The parasitological result in all the survey months (October-December) showed an overall detection rate of 4.4% (48/1082) (CI 95%; 3.2-5.7%) malaria parasite. Among infected individuals, 32 (3.0%) of the infection was due to *Plasmodium vivax *and the rest 16 (1.5%) were due to *Plasmodium falciparum*. The highest prevalence 39(3.6%) of the parasite was observed in age groups of above 15 years old. Among the total tested, 25(2.3%) of males and 23(2.1%) of females had malaria infection. Among tested individuals, 38(5.3%) and 10 (2.7%) of infection was occurred in Misrak-Meskan (2100 m a.s.l) and Mirab-Meskan (2280 m a.s.l), respectively which was statistically significant (X^2 ^= 3.72, P < 0.05). Although the prevalence pattern of *Plasmodium *species declined from October to December, the trend was non-significant (X^2 ^for trend = 0.49, P > 0.05). The entomological survey showed a collection of 602 larvae and 80 adult *Anopheles*. *Anopheles christyi *was the dominant species both in the first (45.3%) and in the second (35.4%) surveys; where as, *Anopheles gambiae sensu lato *comprised 4.7% and 14.6%, in the first and second surveys, respectively. *Anopheles gambiae s.l *comprises 55% of the adult collection, and both species were collected more from outdoors (57.5%). The number of *An. christyi *was higher in Mirab-Meskan (58. 3%) than Misrak-Meskan (41.7%) (P < 0.05).

**Conclusion:**

Malaria parasite and its vectors were found to be common during transmission periods in the highland fringes of Butajira. Thus, health education about the risk of malaria and its control programme in the area must be given adequate attention to minimize potential epidemics. In addition, the current study should be complemented from sero-epidemiological, prospective longitudinal and retrospective studies along with metrological and ecological factors, and socio-demographic data before concluding in favour of highland malaria transmission in the area. In light of its abundance, which coincided with the malaria transmission seasons, the possible role of *An. christyi *as a secondary vector in the highlands must be further investigated by including blood meal sources detection.

## Background

Malaria is one of the world's most serious and complex public health problem. Its transmission is usually associated with topography, climate and socio-economic conditions. The problem of the disease in Africa is aggravated by climate change [1], poverty and lack of efficient controlling mechanism [2]. Also, the emergence of insecticides and drug resistance, human population growth and movement, land use change, and deteriorating public health infrastructures contribute to the spread of the disease [3]. Because of climate and ecological diversity, there is variation in the epidemiology of its transmission. In the stable transmission, the community usually develops herd immunity to the disease; whereas, in unstable form, immunity is absent, and recurrent epidemics are very common [4,5].

Due to Ethiopia's extraordinary diverse topography and climatic conditions, the epidemiology of malaria in the country is more variable and unstable than any other country in Africa. Highland fringe areas at an altitudinal range of 1,500-2,500 metres above sea level (a.s.l.) i.e. northern and central parts and lowland arid areas i.e. eastern and southern parts at altitudinal ranges of below 1,500 m a.s.l. are prone to malaria epidemics. Some areas in western lowland, however, have relatively stable transmissions [6]. A cyclic epidemic with a period of five to eight years occurs in most parts of the country following climatic changes [6]. All age groups are particularly vulnerable to severe malaria during epidemics.

All of the four human malaria parasites species are occur in Ethiopia, but *Plasmodium falciparum *and *Plasmodium vivax *are the dominant species responsible for frequent morbidity and mortality cases. A total of 42 species of *Anopheline *mosquitoes have been documented in the country. The works of Giaquinto-Mira [7], Covel [8], Verrone [9,10] and O'Connor [11] have contributed to the identification and the distribution of malaria vectors in Ethiopia. *Anopheles arabiensis *and *Anopheles quadriannulatus spp *B are the two major species of *Anopheles gambiae *complex that are found in Ethiopia [12] and causing for most malaria transmission.

The conditions and nature of highland malaria transmission of the country were the same to the Butajira highland fringe areas in which epidemics were reported several times [5]. However, unlike in the lowland area, no systematic malaria control programme has been known to be undertaken in Butajira and the highland rural locality in recent years. However, the District is characterized by seasonal malaria, with year to year fluctuations in the number of cases. Moreover, there is no a report from the area whether the appearance of malaria in the high lands of Butajira fringes (Misrak-Meskan and Mirab-Meskan) is due to either endemicity of the parasite or due to high land malaria transmission which might resulted in due to environmental and climate changes. Generally, in Ethiopia, particularly in Butajira highland, there is scanty of information regarding highland malaria transmission. Therefore, the current study aimed to determine the presence and magnitude of malaria transmission from parasitological and entomological data in the highland fringes of Butajira area.

## Methods

### The study area and population

Butajira district is located in Gurage zone, Southern Nations, Nationalities, and Peoples Regional state (SNNPR) of Ethiopia. The District borders includes Siliti zone to the south, the Mareko District to the east, the Muhur and Akillial District to the west, and the Sodo District to the north. The estimated size of the district is 56,200 hectares. The district lies at an average altitude of 1,900 m a.s.l. ranging from 1,750 m a.s.l. in the lowlands to 3,400 m a.s.l. in the mountains. The population of the district is estimated to be 216, 968 in 2005 (Regional Statistical Office). The District has one Hospital, three Health Centers, 32 functional Health Posts, 11 registered non-governmental Health facilities.

Annual rainfall ranges between 700 to 1,870 mm. The main rainy season is from June to September, with light rain common around March and April. The warmest months are between January and June with maximum average temperature of 27.6°C and 27°C in February and March, respectively in the last six years (National Meteorology Service Agency). The area has rich fertile soil and farming is the main mode of living. *Teff (Eragrostos teff)*, maize, millet, barley and legumes are the main crops that the local people produce. Pepper and khat (*Catha edulis forskal*) are also grown as cash crops. Enset (false banana) cultivation is very common and gives the main staple food in the area. Extensive crop cultivation and relatively high population density leave little room for animal husbandry.

The survey was conducted in two *Kebeles *(smallest administrative unit in Ethiopia) in Butajira District, which have altitudes higher than 2,000 m a.s.l. The selection of the two farming associations was not random but based on the prevalence of malaria which was obtained from Butajira Health Center. From all highland localities, Misrak-Meskan was chosen to determine the altitudinal limit at which malaria was prevalent. In addition to a topographical map, a handheld Global Positioning System (GPS) unit was used to record the elevation of the two Farming Associations. Based on this, the average altitude of Misrak-Meskan Farming Association is 2,100 m a.s.l., while the average altitude of two neighboring localities is 2,500 m and 2,600 m a.s.l., respectively which was not included in the study because they were considered to be beyond *Anopheline *flight range. The other study site was Mirab-Meskan, which has the average altitude of 2,280 m a.s.l. The proportion of sampled individuals in the two localities was determined using the size of the proportion of the population living in the area.

### Study design and period

For parasitological surveys, longitudinal study was done from October to December 2006 each month. The entomological surveys were also done from October to December 2006 and continued from April to May 2007. These two separate seasons (from October to December 2006 and April to May 2007) were chosen since sporadic rain which is favourable for vector reproduction is common in Ethiopia before and after the rainy season which is from June to September (8).

### Sampling and sample size determination

Considering 95% confidence interval, 5% margin of error and 40% proportion (previous malaria prevalence obtained from Butajira Health Center) and 5% contingency using the formula for estimating single proportion, a total of 387 patients were included in this study. However, 366 individuals were given eligible data and blood collection was repeated for the three months. Thus a total of 1082 (98.5%) blood films were done and 16 (1.5%) of them was rejected due to various reasons.

### Sampling technique

The lists of households of the two- selected localities were used as a sampling frame, with an assumption of similar exposure of houses and random malaria infections among inhabitants, a total of 100 households were selected systematically based on the calculated proportion for both localities to the sample size of 366 individuals. The calculated proportion was based on the total population of the two localities obtained from Meskan District Administrative office.

If the occupants refused to participate in the survey, the household nearest to it was selected. After obtaining consent, blood samples were taken from all selected family members of the household by fingerpick, and thick and thin smears were prepared. This was done monthly on the same individuals from October through December.

### Blood sample collection in the study area

Blood film collection was performed in peak malaria transmission season of the country [8,13,14] from October to December 2006 after cessation of the major rainy season. A finger prick was done by trained professionals from randomly selected houses by house to house visit. Thin and thick blood smears were prepared on the same slide and allowed to air dry. This was transported to the laboratory where it was stained with Giemsa. Thick and thin films were examined microscopically for the presence of *Plasmodium *and species identification, respectively.

### Entomological survey

Both immature and adult mosquitoes were collected to determine the species composition. Female adult *Anopheles *mosquitoes were used to determine the sporozoites infection rate. Samples were collected from resting sites and also from breeding sites. The collected specimens were identified to species level on morphological basis using Verone [9,10] for both adults and larvae.

### Collections of larvae

Larval collection was performed from suspected breeding sites such as artificial ponds, stream margins, swamps, excavation ditches and rain pools using standard dippers (350 ml) twice a month in a major malaria transmission seasons (October-December) and minor rainy seasons (April-May). The surface area of each potential mosquito breeding site was estimated in square meter (m^2^) and sampling was made at a rate of 6 dips/m^2 ^(four dips at the margin and two from the middle). One 'sample' was defined as 30 dips (or lesser, in smaller sites) taken over a surface area of 5 m^2 ^for sites in the range of 5-10 m^2 ^and forth [15]. Larval *Anophelines *sampled from each type of breeding habitat were transferred to separate vials by direct pipetting. The collected samples were immediately preserved in 75% ethanol. The 3^rd ^and 4^th ^instars larvae then, mounted on slides and identified [10].

### Adult mosquito collection

#### Indoor collections

Adult *Anopheles *mosquitoes, resting inside habitations were collected from selected houses found near to the marshy and other susceptible areas (bush, tall grass, animal shed, in pits, on ground or under logs of wood, artificial ponds, stream margins, swamps, excavation ditches and rain pools). The houses were randomly selected from both localities and at each month, samples were taken twice among five houses in each locality. The collection was done for about 20 minutes each by two persons for two days for every visit. For the second collection, houses selected during first collection were not considered. Therefore, for the five months, a total of 50 houses were selected for mosquitoes collection.

Collection was done during major malaria transmission period through October to December and minor peak in April to May. The collections were done by space spray (knock down collection), CDC light catches and mouth operated aspirators. Space spray collections were conducted early in the morning from 06:00 to 08:00 among selected houses. A white sheet of cloth and aerosol were used to perform space-spray (knock down) collection.

After consent from inhabitants, all occupants, removable house utensils, foods and drinks were removed from the sprayed room. All opening that allowed mosquito escaping, doors and windows were closed: the entire floor was covered with the white cloth sheet. The houses were then sprayed with aerosol for about 10 minutes. After waiting about 10 minutes, after spraying, the sheet was brought outside the room to inspect and collect the fallen mosquitoes. The mosquitoes were picked using forceps and put in box for later identification and for sporozoites rate determination.

Dry cell battery- operated CDC light trap were placed indoors near the bed from 18:00 in the evening to 06:00 in the mooring to collect endophagic *Anopheles *mosquitoes, and then killed with chloroform. Mosquitoes were also collected indoors using aspirator from their resting sites. A flash light was used in locating the mosquitoes in relatively dark parts of the interior of a dwelling. The collected mosquitoes were transferred to a box and killed with chloroform vapor.

#### Outdoor collections

Mosquitoes were surveyed and collected from outdoor resting places among vegetation (bush, tall grass), in animal shed, in pits, on ground or under logs of wood by the help of aspirator. Battery operated CDC light traps were hung in vicinity of sampled houses from 18:00 in the evening to 06:00 in the mooring twice for each month.

#### Sporozoite detection by enzyme-linked immunosorbant assay (ELISA)

The collected female *Anopheles *mosquitoes were dry-stored on silica gel before running the sporozoite ELISA [16]. Two separate 96-well, micro titer plates were coated with 50 μl of the appropriate monoclonal antibodies solutions. For *P*. *falciparum *0.2 μg per 50 μl PBS was used and for *P. vivax *0.025 μg per 50 μl PBS was used. The plates were covered and incubated for 30 minutes after which well contents were dump emptied and filled with 200 μl BB buffer for one hour for further incubation. At the time mosquitoes were ground with 50 μl BB; Igepal 630 with pestle. To avoid contamination between mosquitoes the pestle were cleaned with tissue paper. After removal of buffer, 50 μ l mosquitoes triturate were added to wells of both *P. falciparum *and *P. vivax *duplicates test wells. The test was controlled using positive and negative controls. The control procedures were the same as that of the test samples. Then the plates were covered and incubated for 2 hours and washed 2 times with PBS-tween 20. Fifty micro litter aliquots of homologous peroxidase- conjugated MAB (0.05 μ g/50 μ l BB) were dispensed to each well of respective plates. After one hour incubation the plates were emptied and washed with PBS-tween 20 three times. Hundred micro liter substrates were added to each well after washing. Finally the plates were read visually or by 405-415 nm ELISA reader after adding the substrate. Samples were considered positive if the absorbance values exceeded the mean absorbance of seven negative controls [16].

### Ethical considerations

After discussing the objective and application of the study to Meskan District Health Office and elders of the study area, the inhabitants were contacted to secure their consent in the same way. Blood smear was obtained with finger prick using disposable blood lancet and cotton immersed in 75% alcohol. Treatment of malaria to confirmed cases was given in collaboration with Misrak-Meskan Health Post freely. The study obtained ethical approval from the ethical committee of Biology Department, Addis Ababa University.

### Data analysis

Data were managed and analysed using a statistical computer programme SPPS version 13.0. Simple descriptive statistics and chi-squire tests were used to assess different variables.

## Results

### Socio-demographic characteristics

A total of 366 study participants were enrolled in the study, but in the last survey conducted in December, 2006, 16(4.4%) were missed due to different reasons. The study population was composed of mainly individuals above 15 years old (67.8%). The age groups vary from nine months to 88 years old. Among the total study participants, 188(51.4%) were males and 178(48.6%) were females.

### Parasitological survey

From the three months survey (October, November, and December, 2006) of the study area, malaria prevalence was determined. Out of the total 1082 blood film examined in three month surveys 48(4.4%) (95% CI; 3.2-5.7%) were infected and among these 32(3.0%) and 16(1.5%) were due to *P. vivax *and *P. falciparum*, respectively. *Plasmodium vivax *was the dominant species detected in each of the survey, even if, the difference was not statistically significant (Table 1). Malaria prevalence was decreased from 28 (2.6%) to 18 (1.7%) and then lastly to 2 (0.2%) from October to December, respectively (Table 1) but the trend was non-significant (X^2 ^for trend = 0.49, P > 0.05). The decline through the study months was also evident among Misrak-Meskan and Mirab-Meskan and the sexes (Table 2).

**Table 1:** Prevalence of *Plasmodium *species in the highland fringes of Butajira area from October to December, 2006

Months*	Individuals examined	*Plasmodium *species identified				
				
		*P.falciparum* *N(%)*	*P.vivax N(%)*	Total *N(%)*	X2	P-value
October	366	10 (2.7)	18(4.9)	28 (7.7)	2.38	0.12
November	366	6 (1.6)	12 (3.3)	18 (4.9)	2.05	0.15
December	350	0 (0)	2 (0.6)	2 (0.6)	-	-

	1082	16 (1.5)	32 (3.0)	48 (4.4)		

*X^2 ^for the trend = 0.49, P > 0.05

**Table 2:** Malaria prevalence pattern over the three month period by locality and sex

Month	Individuals examined	Sex			Locality		
		
		Male N (%)	Female N (%)	Total N (%)	Misrak-Meskan N (%)	Mirab-Meskan N(%)	Total N(%)
October	366	16 (4.4)	12(3.3)	28(7.7)	23(6.3)	5(1.4)	28(7.7)
November	366	7(1.9)	11(3.0)	18(4.9)	13(3.6)	5(1.4)	18(4.9)
December	350	2(0.6)	0(0.0)	2(0.6)	2(0.6)	0(0.0)	2(0.6)

**Total**	1082	**25(2.3)**	**23(2.1)**	**48(4.4)**	**38(3.5)**	**10(0.9)**	**48(4.4)**

**Figure 1:**
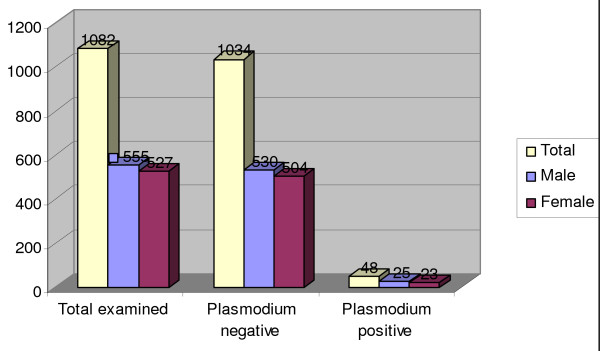
**Frequency of *Plasmodium *species among sexes in the highland fringes of Butajira area from October to December, 2006**.

**Figure 2:**
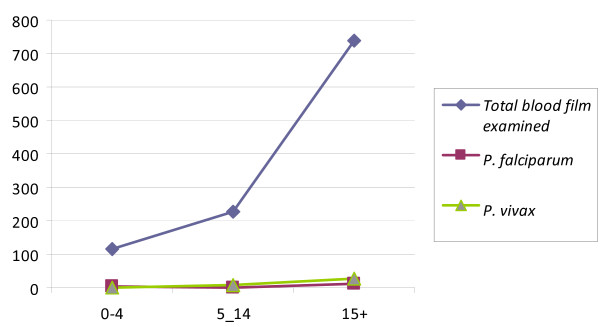
**Prevalence pattern of *Plasmodium *species among different age groups in Butajira highland fringe area from, 2006**.

Among the total tested, 25(2.3%) of males and 23(2.1%) of females had malaria infection (Figure 1). Among all tested individuals, infection was occurred in all age groups and the highest prevalence 39(3.6%) of the parasite was observed in age groups of above 15 years old (Figure 2). Malaria prevalence was observed in the two localities of the study area. Among tested individuals in each locality, 38(5.3%) and 10 (2.7%) of infection was occurred in Misrak-Meskan (2100 m a.s.l) and Mirab-Meskan (2280 m a.s.l), respectively which was statistically significant (X^2 ^= 3.72, P < 0.05) (Table 3).

**Table 3:** Prevalence of *Plasmodium *species among Misrak-Meskan and Mirab-Meskan localities in Butajira highland fringe areas from October to December, 2006

**Locality**	**Average altitude (a.s.l)**	**Total examined** **N (%)**	**Total Positive** **N (%)**	
	
Misrak-Meskan	2100 m	711 (65.7)	38(79.2)	X^2 ^= 3.72, P < 0.05
Mirab-Meskan	2280 m	371(34.3)	10(20.8)	
	
**Total**		**1082(100.0)**	**48(100.0)**	

### Entomological survey

#### Larval mosquito survey

During the study period, a total of 602 *Anopheline *larvae were collected from different types of breading sites. The majority was collected from swamps (32.9%) in Misrak-Meskan, which served as a breeding site during the dry season, while the others were collected in different breeding sites (Table 4). Among the collected 602 larvae, two species were identified, namely *Anopheles christyi *and *An. gambiae s.l. Anopheles christyi *was the most abundant during the first three months survey (45.3%) (October to December) as well as in the second survey (April to May) (35.4%), whereas *An. gambiae s.l *comprised 4.7% in the first survey while it was 14.6% during the second survey season (Table 4).

**Table 4:** Larvae and adult *Anopheles *mosquitoes collected from various breeding sites in Misrak-Meskan and Mirab-Meskan localities in Butajira highland fringe area from October to December, 2006 and from April to May, 2007

Larvae collection		Study locality	Average	*An. gambiae s.l *		*An. christyi *		Total
					
			Altitude (a.s.l)	Oct-Dec	Apr-May	Oct-Dec	Apr-May	
	**Excavations** **Ditches**	Misrak-Meskan	2100 m	23(18.2)	21 (16.7)	44(34.9)	38(30.2)	126 (20.9)
		Mirab-Meskan	2280 m	5 (16.7)	4 (13.3)	14 (46.7)	7(23.3)	30 (5.0)
**Breeding site**	**Rain pools**	Misrak-Meskan	2100 m	0	35(53.8)	0	30(46.2)	65 (10.8)
		Mirab-Meskan	2280 m	0	28 (62.2)	0	17 (37.8)	45 (7.5)
	**Stream margins**	Misrak-Meskan	2100 m	0	0	17 (100.00)	0	17 (2.8)
		Mirab-Mskan	2280 m	0	0	0	0	0
	**Swamps**	Mirab-Meskan	2100 m	0	0	143 (74.2)	55 (27.8)	198 (32.9)
		Mirab-Meskan	2280 m	0	0	55 (45.5)	66 (54.5)	121(20.1)

Total				28 (4.7)	88(14.6)	273 (45.3)	213 (35.4)	602(100.0)
**Adult collection**	*Anopheles *							

	**Misrak-Meskan**	Indoors	2100 m	6(28.6)	8(34.8)	3(25.0)	3(12.5)	20 (25.0)
		Outdoors	2100 m	8(38.1)	6(26.1)	2 (16.7)	7(29.2)	23(28.8)
	**Mirab-Meskan**	Indoors	2280 m	4(19.0)	3(13.0)	2(16.7)	5 (20.8)	14(17.5)
		Outdoors	2280 m	3(14.3.0)	6 (26.1)	5(41.7)	9 (37.5)	23(28.8)

		Total		21(26.3)	23(28.8)	12(15.0)	24 (30.0)	80 (100.0)

During the dry months small pockets of rain pools and associated collection of water disappeared, due to this the swamps and edges of Aufaro River were found to harbor *Anopheline *larvae. The swamp that is found in the study area was a typical breeding site to *An. christyi *whereas *An. gambiae *s.l larvae were collected during the light rainy season in rain pools and in excavation ditches both in October to December and April to May. The larvae of *An. gambiae s.l *and *An. christyi *were collected in different breeding sites at different localities to an altitude range 2,280 m a.s.l. Larger numbers of larvae was collected in Misrak-Meskan than Mirab-Meskan in both species (Table 4), which has a lower altitude (2,100 m a.s.l.).

#### Adult mosquito survey

A total of 80 *Anopheles *species were collected from both indoor and outdoor sites by various methods. The species and the number of *Anopheles *mosquito collected by different methods are also presented in Table 4. Among collected *Anopheles *mosquitoes, *An. gambiae s.l *comprises 55% of the collection while the remaining 45% was *An. christyi*. Both species were collected more (57.5%) from outdoors in both from Misrak-Meskan and Mirab-Meskan communities. The number of *An.christyi *(58. 3%) out number in Mirab-Meskan than Misrak-Meskan (41.7%) (P < 0.05).

#### ELISA sporozoite rate determination

All the collected (80) *An. gambiae s.l *(44) and *An. christyi *(36) adult mosquitoes were assayed by ELISA to determine the infection rate, but none were found to be infected with malaria parasites.

## Discussion

This study determined the presence and magnitude of malaria prevalence and the periodic malaria parasite transmission by parasite specific species, sex and age structures of the population, and the study communities. The mosquito breeding sites, sprozoites rate, and the *Anopheles *species composition in the area were also surveyed.

In Ethiopia, all of the four human malaria parasites species have occurred. However, *Plasmodium falciparum *is the dominant species, accounting for 60-70% of the cases followed by *Plasmodium vivax*, which is responsible for 30-40% of the cases (17). Furthermore, *P.falciparum *is known to be the dominant species during the peak malaria transmission (September and October) while *P. vivax *tends to dominate during the dry season [17]. In the current study, however, *P. vivax *was the dominant cause of infection than *P. falciparum*. The early appearance of the dry season in September 2006 (since the monthly rainfall measurement was less by 268.8 mm from 2005 and by 970.8 mm from 2007) (National Meteorology Service Agency of Ethiopia) and the commencement of data collection in October (more dry than September) may have contributed to the dominance of *P. vivax *during the present survey. Furthermore, the relapsing cases or nature of *P. vivax *may have increased the prevalence of the species in the area during the survey.

The survey also showed that malaria cases declined from October to December 2006. There was also a clear decline in prevalence over the three month period among the locality and sex. This is actually in agreement with the overall malaria epidemiological picture of the country [13]. However, the treatment of positive individuals at the earlier surveys probably contributed to the decline in this survey.

Malaria was observed in both survey localities in the study area, the high number of cases was seen in Misrak-Meskan which might be associated with the presence of swamps near the village and the relatively lower altitude (2,100 m a.s.l.) or the highest test done in the locality may be the sources of bias for higher prevalence record. The swamps that contained water throughout the study period was determined to support *Anopheles *mosquito vector breeding. The two farming associations have numerous water bodies, such as swamps, rivers, and streams. The Rivers Auffaro and Erinzaaf being the major ones. The overflow of Auffaro River is common during the rainy season; due to which numerous breeding sites created during the summer season near villages of Misrak-Meskan locality.

It is also known that in hyper or holoendemic conditions, the occurrence of malaria infection in infants or children in stable communities confirms autochthonous transmission and the standard characterization of the epidemiology of malaria is based on an age-prevalence curve. With the prevalence of infection peaking at an early stage with an increases up on the age of 9 years and showing a sudden fall in the age group 10 to 15 years and then slowly declining with age [18], whereas in populations where malaria is either previously absent or persisted at low or moderate endemic levels, it is characterized by high incidence at all age groups [19]. Such inter age variation in malaria prevalence was reported from Nazareth, Ethiopia [20]. However, the finding from Butajira highland fringe area where age prevalence was not evident would suggest be a relatively recent introduction to the region or could indicate low transmission. Such lack of inter age variation in malaria prevalence in newly introduction malaria has been documented from Jabalpor in central India [21].

In the present study, relatively high malaria prevalence with statistically significance was observed in individuals above 15 year of age. This suggests the poor development of immunity to the malaria parasites in the population. Thus, the individuals aged above 15 years would make up the bulk of the population that probably constitutes the main reservoir of infection in the study area since the dominante species identified was *P. vivax*.

It was also reported that in several places that there has been increased spread of malaria up into highland fringes and the climate change is responsible for this. In particular, global warming was indicated in many reports that it is becoming one of the major causes for encroachment and occurrence of malaria epidemics in high altitude areas of Africa, including Ethiopia [4,22,23]. As some fifty percent of the high land mass in Africa is reckoned to be in Ethiopia [24], the country is considered to be prone to highland fringe malaria. However, there is no metrological and ecological data available to substantiate the current findings in the highlands fringes of Butajira.

The detection of *An. gambiae s.l*. at an altitude of 2,280 m a.s.l was a confirmation of the report Wayessa et al [25] from the outskirts of Addis Ababa, at an altitude of 2,110 m a.s.l. It is also in agreement with the report of Chen et al [26] from the Kenyan highlands. The presence of *An. christyi *in the oily swamps of the study area was in agreement with what was reported from Akaki town where it was found in water bodies polluted by organic substances [26]. Earlier study in Ethiopia has also confirmed that larvae of *An. christyi *unusually tolerate relatively high level of pollution [8]. On the other hand, the oily swamps may have suppressed the distribution of *An. gambiae s.l*., which is the main malaria vector of the country. The abundance of *An. christyi *especially in the highland swamps where malaria is prevalent, makes it suspect as a possible vector of malaria in that locality. However, since *An. christyi *was found to breed in almost all the water collections investigated, its low density in adult collections may be related to its poor anthropophilic behaviour [27], unlike *An. arabiensis *whose feeding behaviour depends on the availability of host, whether outdoors or indoors [28].

The absence of sporozoite infection in the mosquito samples assayed may have been because of the very low sporozoite rate in the mosquito populations in the study area, which had a relatively low level of malaria prevalence. A similar finding has been reported by Abose *et al *[29] regarding the sporozoite detection in *An. arabiensis *in Ziway area, Ethiopia, which gave negative results in both salivary gland dissection and ELISA detection methods. However, the small sample size which was assayed in this study may explain the absence of sporozoite detection.

In conclusion, the parasitological survey showed that malaria parasite was common throughout the survey periods where peak prevalence was seen in the month October, in Mesrak-Meskan community, and in the age groups older than 15 years old. In the entomological survey, *An. gambiae s.l*. and *An. christyi *species were identified. *Anopheles christyi *larvae were the dominant collection whereas the opposite was true in adult collection. Thus, health education about the risk of malaria and its control programme in the upper highland fringes of Butajira must be given adequate attention and recommended to minimize potential epidemics. The sero-epidemiological study as well as assessing the knowledge, attitudes and practices of these high land communities must be done to assess the extent of malaria endemicity or emergence of highland malaria transmission in addition to current study. In light of its abundance, which coincided with the malaria transmission seasons, the possible relevance of *An. christyi *as a secondary vector in the highlands should be further investigated by including blood meal sources detection. In addition, the current findings must be also complemented by prospective longitudinal study and retrospective analysis of documented clinical data in the area. This might help to fill the gaps to the current findings, before concluding in favour of the emergence of highland malaria transmission or due to the endemicity of malaria in the highland of Butajira. Furthermore, the actual cause for the rise in malaria incidence in the area cannot be established without detailed study of changes in climate and agro-ecological factors.

## Competing interests

All authors have declared that no competing interests exist and the manuscript has not been published before or submitted elsewhere for publication.

## Authors' contributions

ST: Conception of the research idea, designing and data collection, data analysis and interpretation, and manuscript reviewing. YB: Data collection, interpretation of the results, and drafting the manuscript. TT: Data collection, interpretation of the results and drafting the manuscript with YB. TM: Designing and data analysis, interpretation, and manuscript reviewing.

BP: Conception of the research idea, designing, data analysis and interpretation, and manuscript reviewing. All authors have read and approved the final manuscript.

## References

[B1] Zhou G Minakawa N Githeko AK Yan G Association between climate and malaria epidemics in East Africa Highland Proc Nat Acad Sci USA 2004 1 2375 2380 10.1073/pnas.0308714100 PMC356958 14983017

[B2] Paul R Climate change and highland malaria in tropics http://www.stabilisation2005.com/poster/Rieter-Paul.pdf

[B3] Hay SI Rogers DG Randpolph SE Stern DI Cox J Shanks GD Snow RW Hot topic or hot air? Climate change and malaria resurgence in East African highlands Trends Parasitol 2002 8 530 534 10.1016/s1471-4922(02)02374-7 PMC3166841 12482536

[B4] Lindsay SW Martens WJM Malaria in the African Highlands: past, present and future Bull World Health Organ 1998 76 33 45 9615495 PMC2305628

[B5] Cox J Craig M Le Sueur D Sharp B Mapping malaria risk in the highland of Africa 1999 Technical Report, Malaria project (MARA/HIMAL). London

[B6] Ministry of Health Federal Democratic Republic of Ethiopic (MOH) Guidelines for malaria epidemic and prevention and control 2004 2 Commercial Printing Enterprise Addis Ababa 52

[B7] Giaquinto-Mira M Notes on the geographical distribution of biology of "*Anophelinae*" and "*Culicinae*" in Ethiopia Imperial Ethiopian Medical Research Institute 1950 9 Addis Ababa 281 313 14809187

[B8] Covell G Malaria in Ethiopia J Trop Med Hyp 1957 60 6 16 13385916

[B9] Verone G Outline for determination of malaria mosquitoes in Ethiopia. Part II. Anopheline larvae Mosquito News 1962 2 394 401

[B10] Verrone G Outline for the determination of malaria mosquitoes in Ethiopia. Part I. Adult female *Anopheles *-mosquitoes in Ethiopia Mosquitoes News 1962 2 38 39

[B11] O'Connor CT The distribution of *Anopheles mosquitoes *in Ethiopia Mosquitoes News 1967 7 42 54

[B12] White GB Tesfaye F Boreham PFL Lemma G Malaria vector capacity of *An. arabiensis *and *An. quadriannulatus *in Ethiopia; chromosomal interactions of after 6 years storage of field preparations Trans R Soc Trop Med Hyg 1980 74 683 684

[B13] Tulu AN Zien Ahemed Zien, Kloos H Malaria The Ecology of Health and Disease in Ethiopia 1993 Westiview Press 341 352

[B14] Fontaine RE Najjar AE Prince JS The 1958 epidemic in Ethiopia Am J Trop Med Hyg 1961 10 795 803 13893940 10.4269/ajtmh.1961.10.795

[B15] Minakawa N Mutero CM Githure JJ Bieber JC Yan G Spatial Distribution and habitat characterization of *Anopheline *mosquito larvae in Western Kenya Am J Trop Med Hyg 1999 61 1010 1016 10674687 10.4269/ajtmh.1999.61.1010

[B16] Wirtz RA Zavala F Charoenvit Y Campbell GH Burkot TR Schneider I Esser KM Beaudoin RL Andre RG Comparatives testing of monoclonal antibodies against *Plasmodium falciparum *sporzoites for ELISA development Bull World Health Organ 1987 65 39 45 3555879 PMC2490858

[B17] Kitaw Y Kelbessa T Gebre-Mariam N The bibliography of malaria in Ethiopia Ethiop J Health Dev 1989 3 57 71

[B18] WHO expert committee on malaria 20^th ^report WHO technical report series 892 2000 Geneva 10892307

[B19] Rudolph H Donato M Quijada I Pena A High prevalence of malaria infection in Amazonas state, Venezuela Rev Inst Med Trop S Paulo 2007 79 79 85 10.1590/s0036-46652007000200003 17505663

[B20] Yohannes M Petros B Urban malaria in Nazareth, Ethiopia: Parasitological studies Ethiop Med J 1996 34 83 91 8840610

[B21] Singh N Chand SK Mishra AK Bahrti PK Singh MP Ahluwalia TP Dash AP Epidemiology of malaria in an area of low transmission in Central India Am J Trop Med Hyg 2006 5 812 816 17123970

[B22] Martens WJM Niessen LW Rotmans JO Jetten TH McMichael AJ Potential impact of global climate change on malaria risk Environ Health Prospect 1995 103 409 525 10.1289/ehp.95103458 PMC1523278 7656875

[B23] Lindsay SW Birley M Climate change and malaria transmission Ann Trop Med Parasitol 1996 90 573 588 9039269 10.1080/00034983.1996.11813087

[B24] Yalden DW The extent of high ground in Ethiopia compared with the rest of Africa Sinet (Ethiopian Journal of Science) 1983 6 35 38

[B25] Woyessa A Gebre-Micheal T Ali A An indigenous malaria transmission in the outskirts of Addis Ababa, Akaki town and its environs Ethiop J Health Dev 2004 81 2 7

[B26] Chen H Githeko AK Zhou G Githure JI Yan G New records of *Anopheles arabiensis *breeding on the Mount Kenya highlands indicate indigenous malaria transmission Malar J 2006 5 17 10.1186/1475-2875-5-17 16522206 PMC1420308

[B27] Gilles MT De Meillon B Publications of the South African Institute of Medical Research No. 54 The Anophelinae of Africa south of Sahara (Ethiopian Zoogrographical Region) 1968 2 Johannesburg

[B28] Ameneshewa B Service MW Resting habits of *Anopheles arabiensis *in river valley of Ethiopia Ann Trop Med Hyg 1996 90 515 521 10.1080/00034983.1996.11813077 8915128

[B29] Abose T Ameneshewa B Tekele-Haimanot A Tulu A Coluzzi M Petrarca V Petrangeli G G Cytogenic studies on *An. gamniae *complex Ethiopia Ethiop J Health Dev 1998 12 81 83

